# An innovative method to strengthen evidence for potential drug safety signals using Electronic Health Records

**DOI:** 10.1007/s10916-024-02070-2

**Published:** 2024-05-16

**Authors:** H. Abedian Kalkhoran, J. Zwaveling, F. van Hunsel, A. Kant

**Affiliations:** 1 Department of Clinical Pharmacology and Toxicology, Leiden University Medical Centre, Leiden, the Netherlands; 2 Department of Pharmacy, Haga Teaching Hospital, The Hague, the Netherlands; 3 The Netherlands Pharmacovigilance Centre Lareb, ‘s-Hertogenbosch, the Netherlands

**Keywords:** Pharmacovigilance, Real World Data, Drug Safety Surveillance, Electronic Health Records, Text-mining

## Abstract

Reports from spontaneous reporting systems (SRS) are hypothesis generating. Additional evidence such as more reports is required to determine whether the generated drug-event associations are in fact safety signals. However, underreporting of adverse drug reactions (ADRs) delays signal detection. Through the use of natural language processing, different sources of real-world data can be used to proactively collect additional evidence for potential safety signals. This study aims to explore the feasibility of using Electronic Health Records (EHRs) to identify additional cases based on initial indications from spontaneous ADR reports, with the goal of strengthening the evidence base for potential safety signals. For two confirmed and two potential signals generated by the SRS of the Netherlands Pharmacovigilance Centre Lareb, targeted searches in the EHR of the Leiden University Medical Centre were performed using a text-mining based tool, CTcue. The search for additional cases was done by constructing and running queries in the structured and free-text fields of the EHRs. We identified at least five additional cases for the confirmed signals and one additional case for each potential safety signal. The majority of the identified cases for the confirmed signals were documented in the EHRs *before* signal detection by the Dutch Medicines Evaluation Board. The identified cases for the potential signals were reported to Lareb as further evidence for signal detection. Our findings highlight the feasibility of performing targeted searches in the EHR based on an underlying hypothesis to provide further evidence for signal generation.

**Supplementary Information:**

The online version contains supplementary material available at 10.1007/s10916-024-02070-2.

## Introduction

Adverse drug reactions (ADR) are one of the substantial causes of hospital admissions and mortality worldwide [1–3]. Therefore, drug safety monitoring plays an important role during the pre- and post-marketing stages of drug development.

Spontaneous reporting of suspected ADRs is the cornerstone of post-marketing surveillance, despite its known limitations [4, 5]. The spontaneous reporting system (SRS) has made an enormous contribution to early and cost-effective detection of ADRs [6–9]. Spontaneous ADR reports arising from the SRS can be hypothesis-generating, however, additional evidence such as more reports are required to further investigate a potential drug-event associations and determine whether it could be considered a safety signal. In practice, there is a considerable time lag between receiving the first spontaneous ADR reports and signal detection, mainly due to under-reporting of adverse drug events (ADEs) which leads to a lack of sufficient evidence. This is while, the required evidence can also proactively be obtained from other real-world sources of information including social media feeds [10, 11], disease and patient registries [12], and patient electronic health records (EHRs) [13–19].

The EHR is an ample source of patients’ health information, stored as structured (i.e. therapies, diagnoses and laboratory test results) and unstructured (i.e. reports and clinical notes) data. Previously, efforts have been made to detect ADEs by searching in single [20] or multiple [21] structured fields of the EHRs. While useful for identifying certain events, this approach offers a limited perspective on a patient’s overall treatment journey and potential ADEs. In reality, a significant portion of relevant patient information lies hidden within the free-text fields of the EHRs, posing a challenge for efficient extraction of information [13, 21, 22]. In recent years, this challenge has been taken up by implementing text mining algorithms for the extraction of the buried information in free-text EHR data [14–19, 23]. Text mining refers to the process of transforming unstructured text into structured data to identify meaningful patterns and models. This is done by combining multiple technologies such as natural language processing (NLP), a subfield of artificial intelligence which allows computer systems to understand text and speech by simulating the human ability to understand a language [24].

The rule-based text-mining software CTcue (IQVIA Patient Finder Solution-CTcue b.v., Amsterdam, the Netherlands) is a novel NLP and text mining based software, which can be used to search on and collect data from the structured as well as unstructured fields of the EHRs. The user-friendly format of this tool has allowed for its widespread use in the Netherlands in the past few years and currently the EHRs of many Dutch hospitals are linked to this software. This software has previously been used and validated to collect RWD on the effectiveness and safety of oncological treatments [25–27].

Different studies have investigated the feasibility of various, often complex, deep learning and NLP tools in EHR-based pharmacovigilance [23, 28–33]. Some have explored hypothesis-free signal detection methods [34] while others have focused on collecting real-world evidence for established ADRs or existing safety signals [29]. While these methods all provide valuable evidence, the EHRs can also provide relevant information in the initial phase of the pharmacovigilance cycle, i.e. prior to signal generation. In order words, the EHRs can be used to identify additional cases for potential drug-event associations based on initial indications from spontaneous ADR reports.

The Netherlands Pharmacovigilance Centre Lareb operates the national SRS. Lareb explores the use of new data sources to complement the SRS, in order to further optimize the process of PV. This study aims to assess the feasibility of identifying additional cases from the EHRs for suspected drug-event associations generated from the SRS in order to strengthen the evidence for the detection of potential safety signals.

## Methods

### Study design

This study is a collaboration between the Netherlands Pharmacovigilance Centre Lareb and Leiden University Medical Centre (LUMC). We selected two confirmed safety signals and two suspected drug-event associations (hereafter referred to as potential safety signals) generated by the SRS of the Netherlands Pharmacovigilance Centre Lareb. Targeted searches were performed in the structured and unstructured fields of the EHR to identify additional cases for these signals using a text-mining software CTcue. The additional cases were then reported to Pharmacovigilance Centre Lareb to be included as further evidence for (potential) signal analysis.

The confirmed signals serve as positive controls in this study. In other words, assuming our employed approach is feasible, we expect to find supplementary cases for the confirmed signals as the drug-event causality has already been verified. The absence of new cases for potential safety signals does not necessarily indicate that our method is infeasible, but rather could be because the potential drug-event associations alluded from spontaneous ADR reports is incorrect.

### Candidate (potential) safety signals

The confirmed safety signals selected for additional case identification were hypokalaemia caused by flucloxacillin use [35] and high anion gap acidosis (HAGMA) caused by the concurrent use of flucloxacillin and paracetamol [36] (Table 1). The signals originated from the SRS and presented to the Dutch Medicines Evaluation Board (MEB) by Lareb in 2015, 2017 (update of previous signal) and 2020. Following discussion at the Pharmacovigilance Risk Assessment Committee (PRAC) of the European Medicines Agency (EMA), both ADRs are listed in the Summary of product characteristics (SPCs) of the corresponding drugs [37, 38].

**Table 1:** The studied confirmed and potential safety signals

**Confirmed signals** **(Established ADRs)**	- Hypokalemia caused by flucloxacillin use [35] - High anion gap acidosis (HAGMA) caused by the concurrent use of flucloxacillin and paracetamol [36]
Potential signals (Suspected ADRs)	- Atraumatic splenic rupture associated with direct oral anticoagulants (DOACs) - Renal failure and acute tubular necrosis induced by ibrutinib

Two potential signals were also selected: atraumatic splenic rupture associated with direct oral anticoagulants (DOACs) and renal failure and acute tubular necrosis induced by ibrutinib (Table 1). Both signals were generated by Lareb during case-by-case analysis and deemed to have sufficient signal strength to be considered a safety signal [39]. These potential signals were discussed with the MEB in separate signal detection meeting in 2020 and 2021 respectively. However, due to a limited number of reported cases, no official signal was issued by Lareb and no regulatory actions were taken by the MEB at the time of the study. As a result, these two potential signals remain candidates for further analysis.

### Data source

All the data used in this study were extracted from EHRs of patients receiving treatment at the LUMC. The LUMC inpatient health records have been fully digitalised since 2011 and are documented in one of the major EHR systems in the Netherlands, HiX (ChipSoft, Amsterdam, the Netherlands). This system contains, among others, data on patient demographics, health behaviour, vital status, laboratory reports, medication prescriptions, procedures, imaging, health problem lists, and free-text notes and reports of the healthcare professionals.

### Process of case-search

**Fig. 1:**
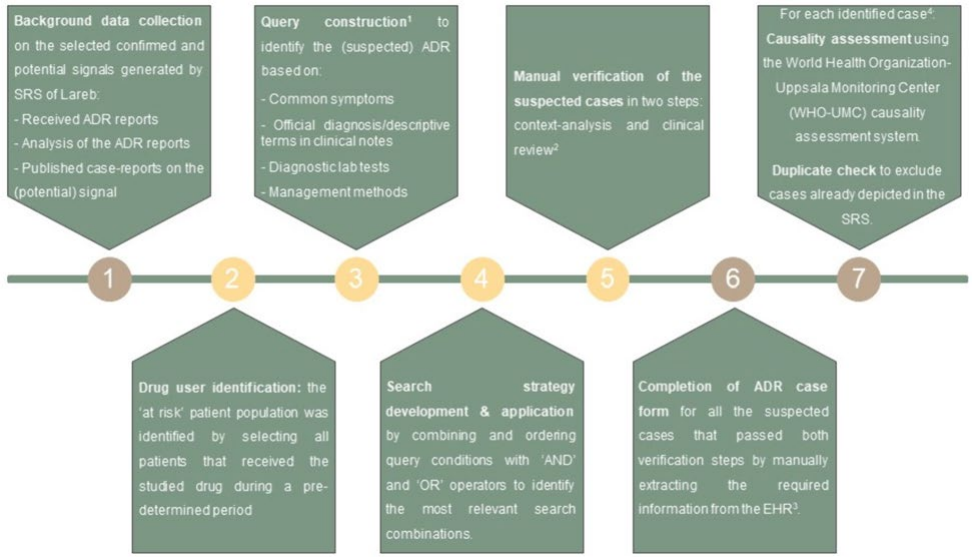
A step by step work plan for case identification; Yellow circles depict the steps that involved using the text-mining software, CTcue

An overview of the process of case identification is shown in Fig. 1.

1. Each query was made up of multiple subqueries and had a, specific, predefined time window.
2. The identified potential cases were manually verified in two steps. First, within the text-mining software, the query keywords were reviewed in context (i.e. context-analysis). Second, the relevant EHR sections of the remaining potential cases were clinically reviewed. The remaining cases, were selected as “suspected cases”.
3. In case of the confirmed signals, the ADR forms were only filled out for five potential cases.
4. The causality assessment and duplicate check were performed by a PV expert of Lareb

Search for potential cases was performed using a text-mining software CTcue (IQVIA Patient Finder Solution-CTcue b.v., Amsterdam, the Netherlands). This tool is linked to the EHRs and allows for an efficient extraction of structured (including medication prescriptions, laboratory results) and unstructured (medical reports, letters and notes) data. All data on drug exposure, experienced ADR and patient-related factors were retrieved under the General Data Protection Regulation (GDPR) in a pseudonymized fashion. The architecture of the text-mining tool is discussed by van Laar et al. 2020 [25].

We performed targeted searches in the structured EHR data by creating systematic queries for each data type. Unstructured EHR data was retrieved through text mining using keywords. To enhance the effectiveness of the keyword-based search, we utilised the text-mining software's built-in keyword library, which incorporates commonly used synonyms, typographical errors, colloquial alternatives, and abbreviations related to the manually generated keywords. Furthermore, to improve search accuracy, we excluded undesired words or word combinations, including negations. Table S1 gives an overview of the created queries for the confirmed and potential drug safety signals under investigation.

The identified potential cases were verified in two steps, after which an ADR case form was filled out for the remaining “suspected cases”. Lastly, a causality assessment and duplicate check was performed by a PV expert of Lareb for each identified case (Fig. 1).

## Results

A summary of the search strategy and findings for each signal is presented in Table 2.

**Table 2:**
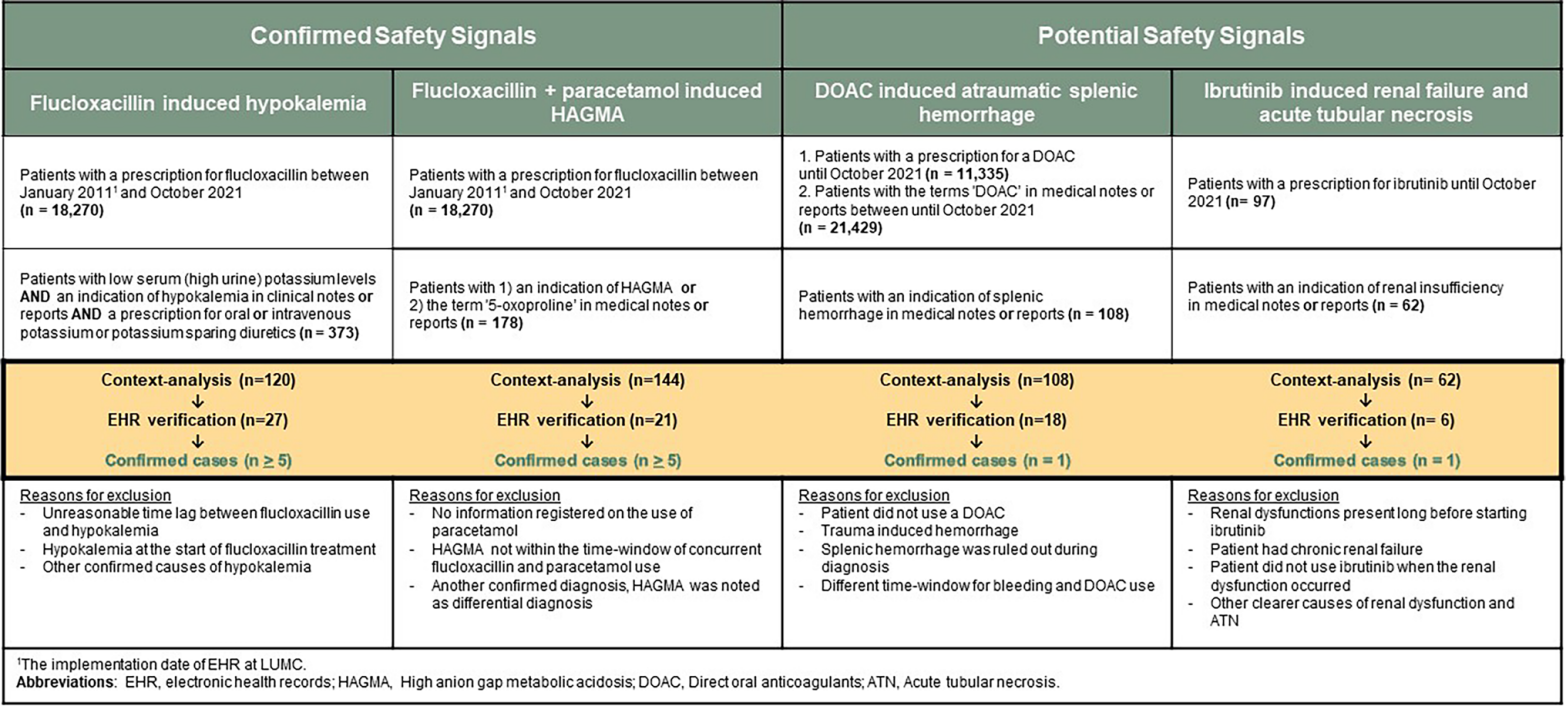
Results of the case identification steps for the confirmed and potential signals. The number of confirmed cases per signal is shown in green.

### Case identification for the confirmed safety signals

#### Flucloxacillin induced hypokalaemia

We identified 18,270 patients who received flucloxacillin between January 2011 and October 2021, among whom 373 patients with low serum (or high urine) potassium levels *AND* a diagnosis of hypokalaemia in diagnosis field, clinical notes *or* reports *AND* a prescription for oral or intravenous potassium or potassium sparing diuretics. Since this signal is already confirmed and case identification is merely to test the feasibility of the study, context-analysis was only performed for a random sample of 120 cases from the 373 found cases. This led to the exclusion of 77.5% (n = 93) of the cases. The main reasons for exclusion include: unreasonable time lag between flucloxacillin use and hypokalemia, low potassium levels at the start of treatment with flucloxacillin and other confirmed causes of hypokalaemia. The remaining 27 patients suspected with flucloxacillin induced hypokalaemia were further verified manually and the ADR forms were filled out until five confirmed cases of flucloxacillin induced hypokalaemia were identified. All the identified cases pertain to the period between 2016 and 2018, before this signal was detected by Lareb in 2020, leading to an SmPC update after discussion at PRAC.

#### HAGMA associated with concurrent use of flucloxacillin and paracetamol

To identify potential cases with high anion gap metabolic acidosis (HAGMA) caused by concurrent use of flucloxacillin and paracetamol, all the patients receiving flucloxacillin (n = 18,270) were selected. Among them, 88 patients had an indication of HAGMA in their clinical notes *or* reports. Additionally, in the clinical notes *or* reports of 90 patients the term “5-oxoproline” was used. The elevation of 5-oxoproline is characteristic for HAGMA induced by concomitant use of flucloxacillin and paracetamol. Context analysis was performed for 144 cases, leading to the exclusion of 85% (n = 123) of the patients, primarily due to absence of paracetamol in the medication list. EHR verification was performed for the remaining cases until five confirmed cases of flucloxacillin and paracetamol induced HAGMA were identified. Three of the five identified cases were documented in the EHRs before signal detection by Lareb in July 2015.

### Case identification for potential safety signals

#### Atraumatic splenic haemorrhage associated with DOAC use

No patients were identified with a DOAC prescription in the medication list and splenic hemorrhage in the EHRs. However, due to the under-documentation of DOAC prescriptions in the inpatient setting, additional queries were created to identify patients with the term ‘DOAC’ (including all the individual [brand] names of the drugs) in the free-text notes (i.e. clinical notes or reports) of the EHRs. Subsequently, patients with splenic hemorrhage were identified based on, among others, the diagnosis terms in clinical notes or reports. After verification, one suspected patient was found to have DOAC induced atraumatic splenic haemorrhage. DOAC use was only recorded in the admission letter of that patient and it was not documented in the medication list.

#### Ibrutinib induced renal failure and acute tubular necrosis

Ibrutinib was prescribed for 97 patients. Following treatment initiation, 62 patients were detected with a mention of ‘renal failure’ in clinical notes or reports. In the first verification step 56 patients were excluded due to chronic renal failure and other confirmed causes of acute renal failure. The clinical notes of the remaining six patients were manually reviewed, which led to the identification of one patient with acute renal failure as a result of ibrutinib use.

### Case assessment and duplicate check

The duplicate check showed that none of the identified cases were previously reported to the SRS. In addition, the causality assessment demonstrated a “probable” or “possible” causal relationship between the ADRs and the corresponding drugs for all identified cases. The identified cases for the potential signals were reported to Lareb and were included as additional evidence for further analysis.

## Discussion

In this feasibility study, we explored the value of EHRs to identify additional cases for potential safety signals generated by the SRS using a relatively simple text mining technique. We identified at least five supplementary cases for two confirmed signals, most of which were documented in the EHRs prior to the approval of the causal association by the Dutch MEB. Furthermore, targeted searches in the EHRs, identified one additional case for each of the two potential signals. These cases were reported to the Pharmacovigilance Centre and included in the Lareb database, thus providing further evidence for signal detection. We acknowledge that the number of identified cases for these potential safety signals is relatively small. However, it should be taken into consideration that these safety signals pertain to rare events (i.e., atraumatic splenic hemorrhage) and involve medications with a limited user population (i.e., 97 patients receiving ibrutinib). Although the initial generation of false positive hits may appear daunting, it is important to highlight that the process of context analysis is highly time efficient. Within a few hours, hundreds of cases can be thoroughly reviewed.

To the best of our knowledge, this is one of the few studies that attempts to strengthen the evidence for potential signals by proactively searching in the EHRs for additional cases based on initial indications from spontaneous ADR reports [40]. Performing targeted searches in the EHR based on an underlying hypothesis is a more direct and efficient strategy for implementing real-world data for pharmacovigilance purposes than hypothesis-free signal detection from EHRs. Other approaches involving EHRs, like pharmacoepidemiological studies and rapid query analysis.

Additionally, unlike other studies that rely on building complex NLP algorithms [41, 42], we employ a relatively efficient and straightforward method to extract data from the EHRs. Furthermore, given the accessibility of our utilized NLP tool in the Netherlands, it has the potential to be further developed and implemented at a national level to complement the existing SRS.

Hospitals commonly conduct medication reconciliation upon admission to ensure an accurate and updated record of patients' medications. Ideally, all home and over-the-counter (OTC) medications should be listed in the structured field of "prescriptions" in the EHR. However, this process is susceptible to errors, leading to medication discrepancies. Generally, the use of OTC medications is better documented when continued during hospital admission. Furthermore, there are cases where home medications are more thoroughly documented in free-text clinical notes rather than in medication lists. One example is the registration of anticoagulant use in EHRs for patients with hemorrhage, as these medications are often discontinued upon hospital admission to prevent further bleeding. In the case of DOAC-induced splenic hemorrhage, initially, no potential cases could be identified when the queries were filtered solely based on DOACs listed in the medication list. However, by performing a keyword search for "DOAC" (including synonyms) within free-text notes, one potential case was found, with apixaban listed as a home medication in a medical report.

The constructed queries are generally filtered to only search within a predefined risk-window relative to the start- and end-date of the investigated medication. However, this can be quite challenging when multiple prescription requests with different start- and end-dates are recorded for the same medication. Examples include medications that are prescribed in courses (e.g. antibiotics including flucloxacillin). The text-mining software is only able to use the date of one prescription (either first or last) as the point of reference for other queries. This limitation turned out to be one of the main reasons for excluding the identified cases during the context-analysis.

The extraction of laboratory results posed several challenges in relation to the studied signals, despite their importance as a diagnostic step. Firstly, the queries were unable to filter results based on sex-specific reference ranges for laboratory tests. Secondly, clinically insignificant short-term deviations from the defined reference ranges resulted in many redundant cases. Thirdly, it was not possible to differentiate between chronic and acute-on-chronic laboratory abnormalities during query design. This distinction was particularly critical for the signal of ibrutinib induced renal dysfunction, as a significant number of patients already had pre-existing chronic renal dysfunction prior to initiating ibrutinib therapy. Consequently, the search results from these queries yielded a considerable number of falsely identified cases, which were subsequently excluded during the context analysis phase.

Queries concerning symptoms, laboratory results and treatments for AEs were found to contribute only minimally to final case identification, since the search results of these queries were very nonspecific. On the other hand, we observed that queries constructed based on diagnostic terms generated the most specific search results for all four signals. These terms included either the name of the event (including all the possible ways to describe it) or a specific feature or diagnostic test (e.g. 5-oxoproline) in clinical notes or reports. Therefore, free-text data searches became the core of case identification in this study.

We also observed that case identification for rare events (e.g. splenic hemorrhage), caused by a commonly used drug, can best be performed by initially identifying all patients with that specific AE and then detecting the users of the specific drug among them.

A key strength of this study is the implementation of a novel, and easy-to-operate, text-mining tool capable of searching for PV information within both the structured and unstructured fields of EHRs. Currently, this tool is exclusively available in the Netherlands, but it is expected to expand its availability to other European countries, along with other text mining tools. An important advantage of this tool is its versatility, as the constructed queries can be reused in different EHR databases. By including confirmed and potential signals involving various types of medications (in-patient vs. out-patient, different levels of monitoring, etc.) and diverse adverse event characteristics (rare vs. common, specific vs. general), we were able to gain a comprehensive understanding of the scope and feasibility of targeted EHR searches for case identification. To minimize the risk of self-fulfilling prophecy bias, we also performed a causality assessment for all the identified cases to specifically evaluate the strength of the cases [43]. For this assessment we included all the information regarding relevant comedications and comorbidities of the suspected cases, as well as the time lag between treatment initiation and the incidence of the adverse event. This approach ensured a rigorous and unbiased evaluation of the relationship between treatments and ADEs.

Yet, our proposed approach also has some limitations. First, accessing patient data in the EHRs for PV purposes is a great challenge due to the strict policies regarding patient privacy. Second, this approach was only tested on one EHR system while the quality of the EHR data may vary between hospitals. Third, strengthening potential safety signals using the data from the EHRs is probably mainly feasible for drugs that are mainly prescribed in the secondary care settings and/or events that occur during hospital admissions or require hospitalization. Therefore, cases involving mild symptoms might not be captured by this approach. Forth, the generated potential cases had to be manually verified before final assessment. Noteworthy is that the process of context-analysis allows for an extremely efficient removal of the majority of the false positive hits and only a small percentage of the generated hits have to be clinically reviewed in the EHR. Finally, the confirmed safety signals served as positive controls in our study due to their established evidence regarding drug-event causality. However, it is important to note that complete certainty regarding their safety profile is elusive. Nonetheless, we believe that the confirmed signals we selected were reasonably chosen given the available evidence.

The results of this feasibility study may be considered an important starting point for our proposed approach. The text-mining tool employed in this study, is currently operational in over thirty hospitals across the Netherlands. Our ultimate goal is to identify additional cases from EHRs in real-time, for potential safety signals that have emerged from spontaneous ADR reports. These identified cases could provide further evidence to generate a signal. Moreover, the ability to share and execute queries in other hospitals equipped with this tool, as well as the convenience of re-running queries at various time points with minimal effort, further illustrates the potential of this approach. Based on our current findings, we believe that this method may particularly be valuable for detecting rare but serious potential ADEs. However, further research is required to explore the generalizability of this approach to other types of potential ADEs, such as those with a delayed onset or less pronounced symptoms. Additionally, other challenges must also be addressed, such as ensuring query portability between institutions both within and outside the Netherlands, and evaluating the value added by the identified additional cases in terms of efficiency and workload for signal detection. Lastly, the practicality of implementing this approach in routine clinical practice needs to be explored.

## Conclusion

We propose a novel approach to proactively utilize patient data from EHRs in order to strengthen the evidence for potential drug safety signals generated by the SRS. The findings highlight the feasibility of applying text-mining to the structured and free-text fields of the EHRs to identify and report additional cases for potential safety signals. This process can greatly contribute to and accelerate signal detection process.

## Availability of data

The dataset for this manuscript is not publicly available because of the Lareb data protection policy. Requests to access the datasets should be directed to the first author and will be granted upon reasonable request.

## Supplementary Information

Below is the link to the electronic supplementary material.

Supplementary file1 (DOCX 20 KB)

## Data Availability

The dataset for this manuscript is not publicly available because of the Lareb data protection policy. Requests to access the datasets should be directed to the first author and will be granted upon reasonable request.
