# Supplementary material for: An innovative method to strengthen evidence for potential drug safety signals using Electronic Health Records
Source: J Med Syst. 2024 May 16;48(1):51. doi: 10.1007/s10916-024-02070-2 (PMC11098892; doi:10.1007/s10916-024-02070-2)
Supplement: Supplementary file 1 — Supplementary file1 (DOCX 20 KB) [file 10916_2024_2070_MOESM1_ESM.docx]

|  | **Query** | **Collected answer** | **type of data extraction** | **Searched terms (in Dutch)** | **Keyword search restricted to** | **Time window** |
| --- | --- | --- | --- | --- | --- | --- |
| **Signal 1: flucloxacillin induced hypokalemia** | Hypokalemia lab result | Measurement | numerical value in mmol/L (structured) | Kalium (SD) <3,5 mmol/L and >125 mmol/L | NA | After first prescription start date flucloxacillin |
|  | Diagnosis hypokalemia | The combination of two diagnosis terms in medical notes and forms | Keyword in free-text (unstructured) | From keyword synonym library: Flucloxacillin Manually added: Flucloxacilline antibioticum antibiotica fluc flucl fluclo fluclox I.C.W From keyword synonym library: Hypokaliëmie hypokaliaemie hypokalemia kaliumtekort lage kalium daling kalium verlaagde kalium | within clinical notes: Policy, conclusions, summary, Medical history, disease course | After first prescription start date flucloxacillin |
|  | Treatment hypokalemia | Medication request | Prescription start date | kaliumchloride Kaliumcitraat | NA | After first prescription start date flucloxacillin |
|  |  |  |  | Triamtereen Spironolacton Eplerenon |  |  |
| **Signal 2: flucloxacillin and paracetamol induced HAGMA** | HAGMA symptoms | Symptoms associated with HAGMA in medical notes and forms | Keyword in free-text (unstructured) | Any of the following terms from keyword synonym library: Benauwdheidsgevoel (+ 5 synonyms) Respiratoire Insufficiëntie (+ 7 synonyms) Kussmaul-ademhaling (+8 synonyms) Ademhalingsdepressie (+17 synonyms) | NA | After first prescription start date flucloxacillin |
|  | HAGMA diagnosis 1 | The combination of two diagnosis terms in medical notes and forms | Keyword in free-text (unstructured) | From keyword synonym library: Metabole acidose Metabool acidose metabolic acidosis acidose metabool acidose I.C.W Anion Gap Verhoogd Hoge Anion-gap Hoge aniongap verhoogde anion gap verhoogde aniongap verhoogd anion gap verhoogd aniongap | within clinical notes: Policy, conclusions, summary, Medical history, disease course | After first prescription start date flucloxacillin |
|  | HAGMA diagnosis 2 | 5-oxoproline in medical notes and forms | Keyword in free-text (unstructured) | From keyword synonym library:  5-oxoproline 5-oxoprolinurie 5-oxo Oxoproline pyroglutamatic acidose oxyproline pyroglutamaat-acid pyroglutamaat acidose Pyroglutamaat-acidurie 5-oxoprolineconcentratie | NA | After first prescription start date flucloxacillin |
|  | HAGMA lab results | Measurement | numerical value in mmol/L (structured) | Bicarbonaat < 24 mmol/l | NA | After first prescription start date flucloxacillin |
|  |  |  |  | Anion gap >12 mmol/l |  |  |
|  | Treatment HAGMA | Medication request | Prescription start date | kaliumchloride Kaliumcitraat | NA | After first prescription start date flucloxacillin |
|  |  |  |  | Acetylcysteine |  |  |
|  |  |  |  | Natriumbicarbonaat |  |  |
| **Signal 3: DOAC induced spontaneous splenic hemorrhage** | Splenic hemorrhage symptoms | Symptoms associated with splenic hemorrhage in medical notes and forms | Keyword in free-text (unstructured) | From keyword synonym library: Buikpijn abdominale pijn Pijn in milt/abdomen Malasie Tekenen van shock Hemodynamische instabiliteit Hemodynamisch instabiel intra-abdominaal vrij vocht Daling Hb | NA | NA |
|  | Splenic hemorrhage diagnosis | Common terms used to diagnose splenic hemorrhage | Keyword in free-text (unstructured) | From keyword synonym library: miltbloeding/bloeding milt/milt bloeding miltruptuur/milt ruptuur/ ruptuur milt/ geruptureerde milt Milthematoom/milt hematoom/ hematoom milt Bloed rond de milt Miltlaceratie/ laceratie in de milt | NA | NA |
|  | Lab results | Measurement | numerical value in mmol/L (structured) | Hemoglobine/Hb < 7.5 mmol/l | NA | NA |
|  | Treatment options | Surgeries | Indication | Milt splenectomie miltexpiratie |  | NA |
|  |  | Fluid IN/OUT | Description | Bloed IN |  |  |
|  |  | Medication request | Prescription start date | protrombinecomplex |  |  |
|  |  |  |  | Plasma versbevroren |  |  |
|  |  |  |  | Factor VIIa |  |  |
| **Signal 4: ibrutinib induced renal dysfunction** | Diagnosis | Common terms used to diagnose renal dysfunction | Keyword in free-text (unstructured) | From keyword synonym library: Nierinsufficiëntie Nier Insufficiëntie Renale Insufficiëntie nierfalen  Tubulusnecrose  Tubulus necrose ATN Verminderde Nierfunctie  nierfunctiestoornis |  | After first prescription start date flucloxacillin |
|  | Lab results | Measurement | numerical value in umol/L (structured) | creatinine/creat > 90 umol/l |  | After first prescription start date flucloxacillin |
|  |  |  | numerical value in ml/min/1,73m2 (structured) | eGFR CKD-EPI/MDRD <60 ml/min/1,73m2 |  |  |
